# Supplementary material for: Garlic Volatile Diallyl Disulfide Induced Cucumber Resistance to Downy Mildew
Source: Int J Mol Sci. 2021 Nov 15;22(22):12328. doi: 10.3390/ijms222212328 (PMC8625977; doi:10.3390/ijms222212328)
Supplement: Supplementary file 1 [file ijms-22-12328-s001.zip › Supplementary Figure.pdf]

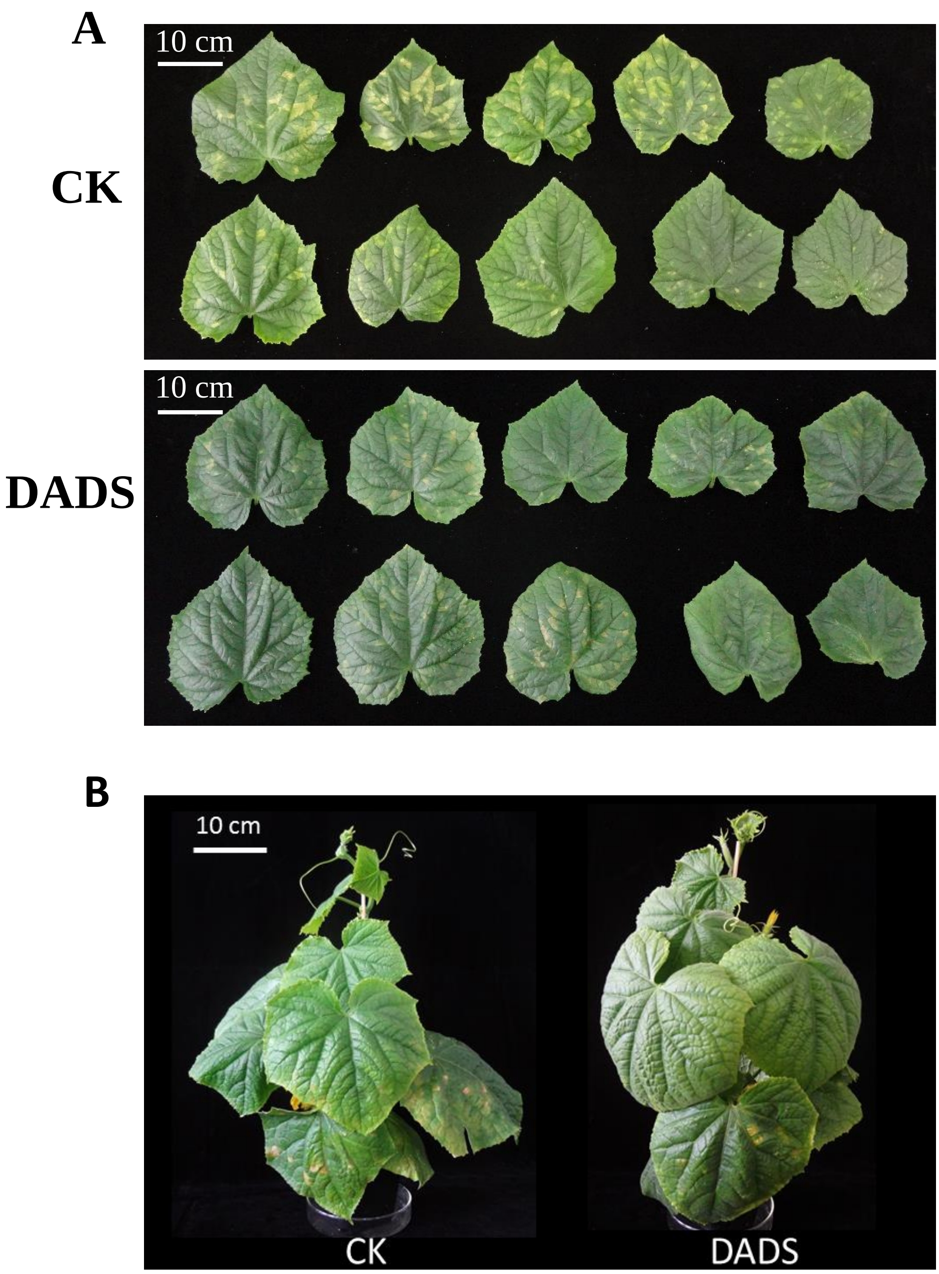

Supplementary Figure S1. Compares in cucumber phenotype of DADS group and CK group. (A) 7 days of inoculation, (B) 14 days of inoculation.

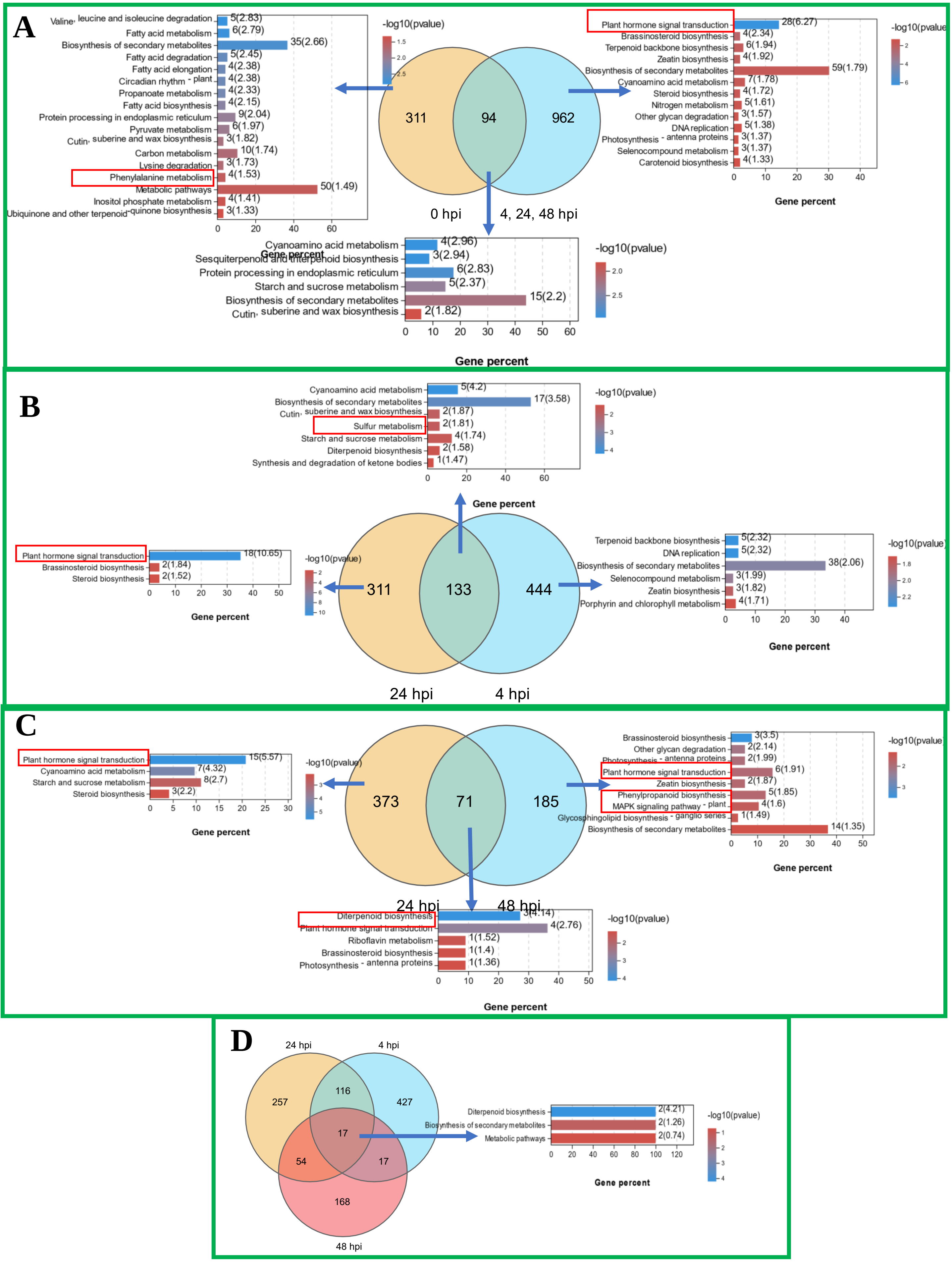

Supplementary Figure S2. Venn analysis and KEGG enrichment analysis of up-regulated DEGs.

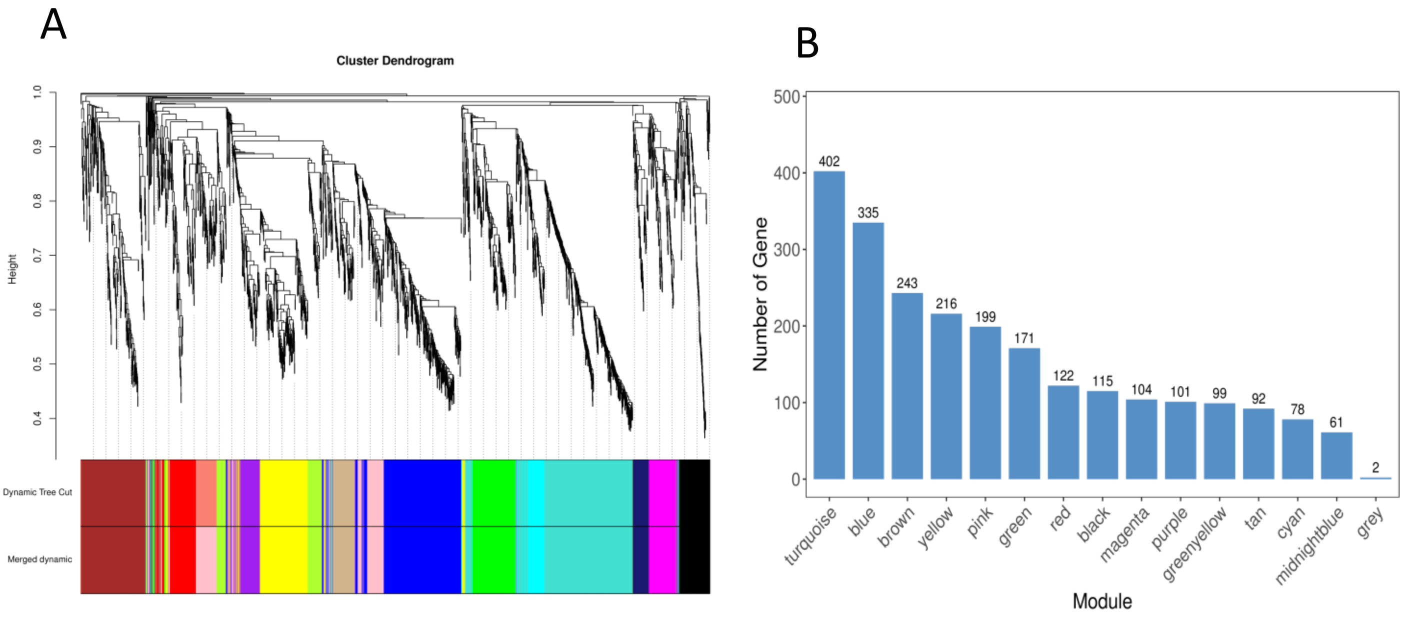

Supplementary Figure S3. The decision of network analysis dendrogram showing modules and module gene numbers identified by WGCNA of up-regulated DEGs. (A) Dendrogram plot with color annotation. (B) Gene numbers of every module.

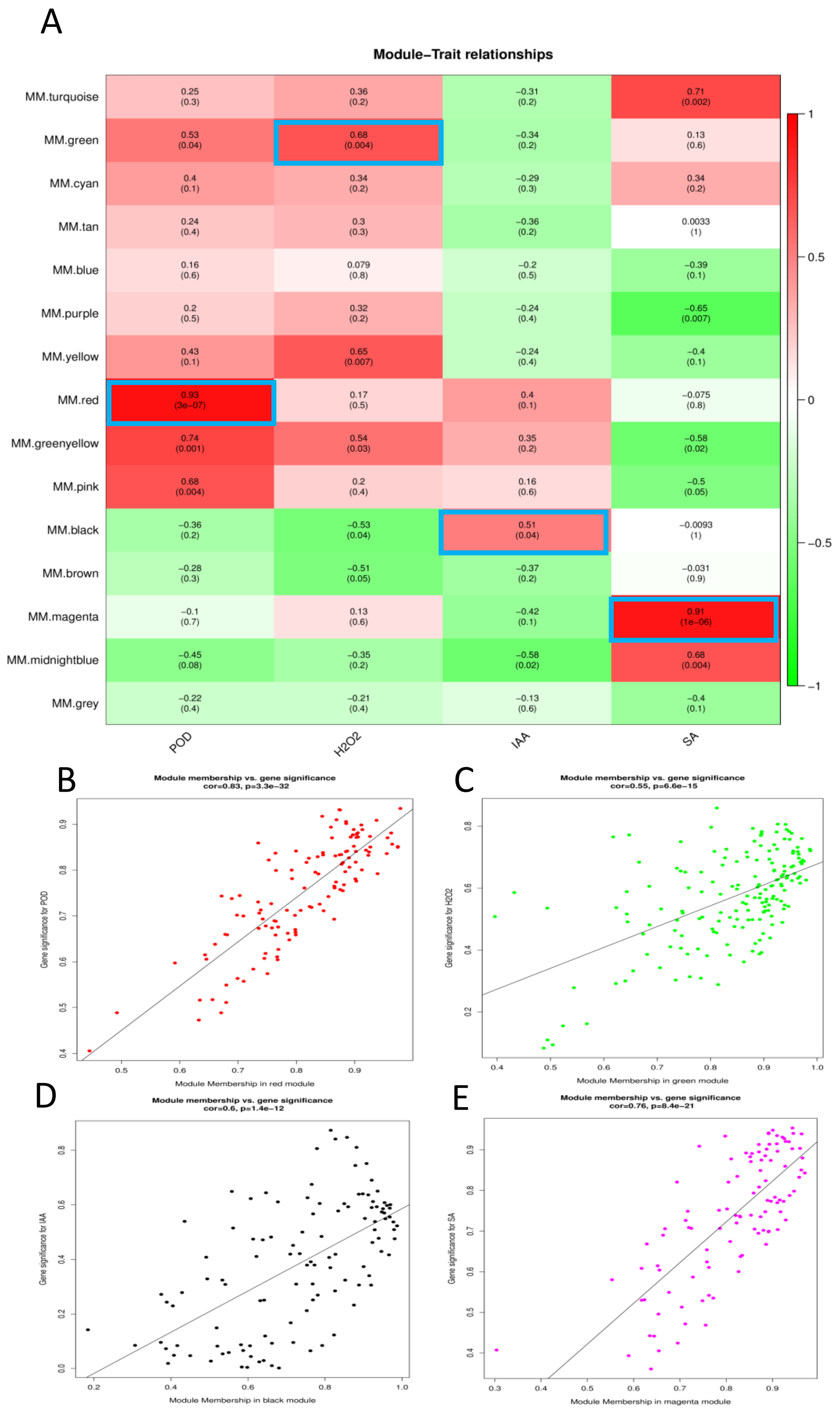

Supplementary Figure S4. Module-trait weight correlations and corresponding P-values, MM and GS relation for modules by WGCNA.

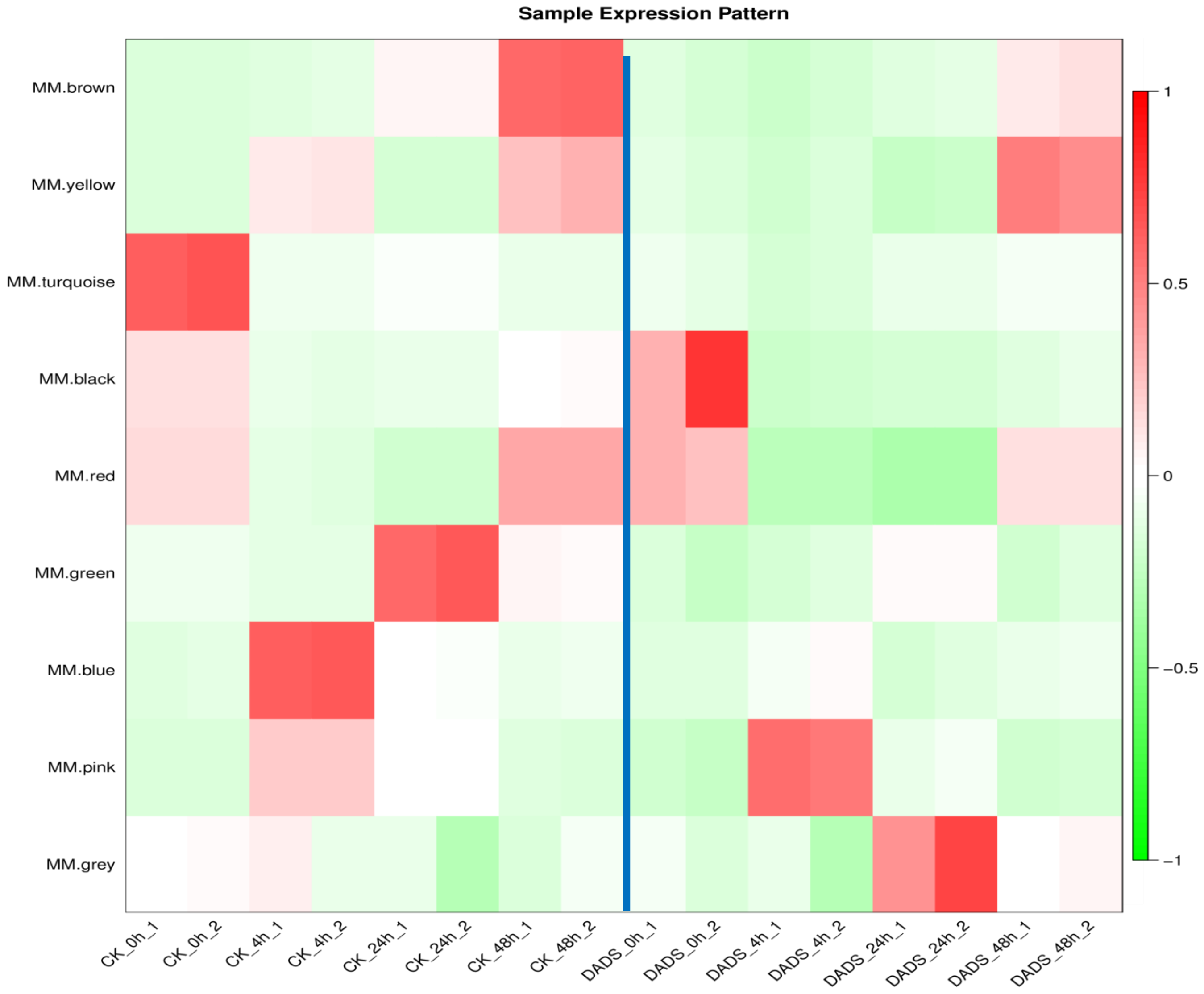

Pathway	Module		
Phenylpropanoid biosynthesis	pink		
Plant hormone signal transduction	blue	green	pink

Supplementary Figure S5 . Expression modules constructed WGCNA and associated KEGG of down-regulated DEGs in the pink’, ‘green’, ‘blue’ modules.

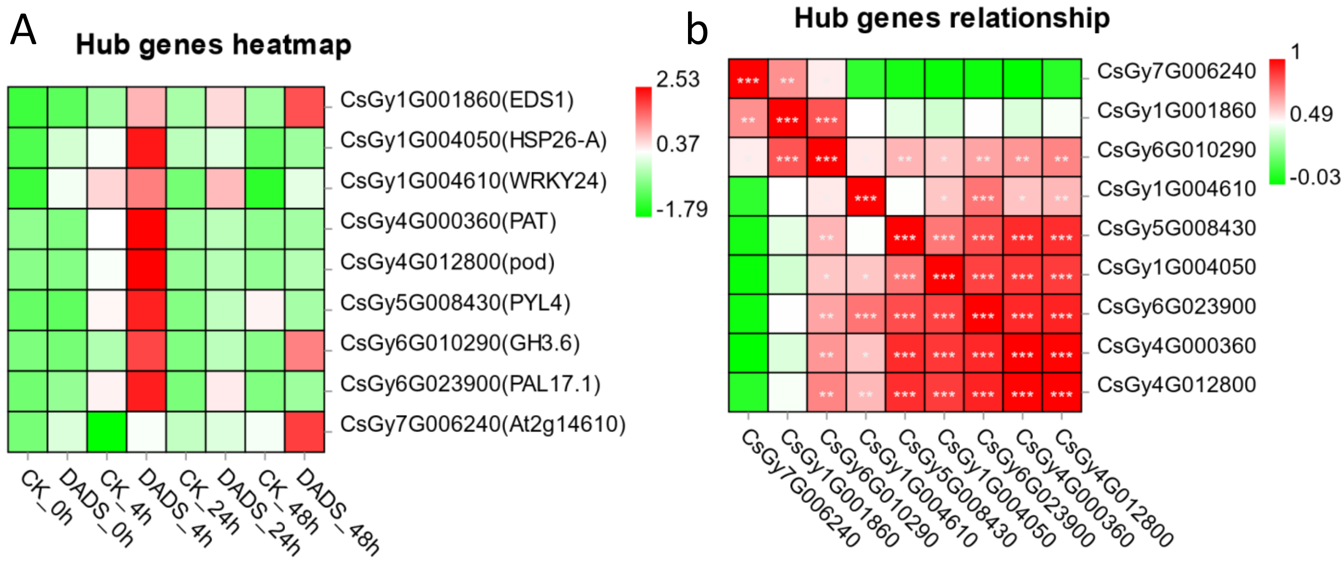

Supplementary Figure S6. Hub genes expression (A) and correlation analysis (B) in modules.
